# DADA2 as a Model of Monogenic Immune Vasculopathy: From Immunopathogenesis to Precision Therapeutics

**DOI:** 10.3390/biom16071057

**Published:** 2026-07-19

**Authors:** Hao Peng, Chunxia Li, Chune Mo, Bihui Li, Minglin Ou

**Affiliations:** 1 Laboratory Center, Guangxi Health Commission Key Laboratory of Glucose and Lipid Metabolism Disorders, The Second Affiliated Hospital of Guilin Medical University, Guilin 541199, China; 2 Genetics and Cellular Engineering Group, Research Unit Signaling and Translation, Helmholtz Zentrum Munich, Ingolstaedter Landstr. 1, 85764 Neuherberg, Germany; 3 Laboratory Center, Guangxi Key Laboratory of Metabolic Reprogramming and Intelligent Medical Engineering for Chronic Diseases, Guilin Medical University, Guilin 541199, China; 4 Department of Oncology, The Second Affiliated Hospital of Guilin Medical University, Guilin 541199, China

**Keywords:** DADA2, adenosine deaminase 2, monogenic vasculitis, PAN, TNF inhibitor, HSCT, genotype–phenotype correlation, precision medicine

## Abstract

Deficiency of adenosine deaminase 2 (DADA2) is a monogenic autoinflammatory disorder caused by biallelic loss-of-function mutations in the *ADA2* gene (formerly *CECR1*). First described in 2014, DADA2 has emerged as a paradigm for monogenic vasculitis, bridging the gap between primary immunodeficiencies and systemic vasculitides. The disease is characterized by a remarkably broad clinical spectrum encompassing early-onset lacunar stroke, systemic vasculitis resembling polyarteritis nodosa (PAN), hematologic abnormalities ranging from pure red cell aplasia to pancytopenia, humoral immunodeficiency, and variable lymphoproliferation. ADA2, predominantly secreted by myeloid cells, serves dual functions as a growth factor for endothelial cells and a modulator of extracellular adenosine metabolism. Its deficiency leads to a proinflammatory state driven by macrophage dysregulation, excessive tumor necrosis factor (TNF) production, neutrophil extracellular trap (NET) formation, and endothelial dysfunction. The genotype–phenotype correlation is complex, with certain mutations predisposing to vasculitic versus hematologic-predominant phenotypes. Emerging evidence further links ADA2 deficiency to cellular senescence and inflammaging pathways, suggesting a connection between monogenic vasculitis and aging-related biological mechanisms. Anti-TNF therapy has revolutionized disease management, achieving sustained remission in the majority of vasculitic manifestations. Hematopoietic stem cell transplantation (HSCT) offers a definitive cure for severe hematologic disease, while gene therapy approaches are under active investigation. This review synthesizes current knowledge on the immunopathogenesis, clinical heterogeneity, genotype–phenotype correlations, multi-omics insights, and evolving precision therapeutic strategies for DADA2, positioning it as an instructive model for understanding monogenic immune vasculopathy. Despite this progress, fundamental questions remain—including the relative contribution of ADA2 enzymatic versus growth factor functions to disease pathogenesis, the mechanisms underlying tissue-specific vulnerability, the basis of differential treatment responsiveness, and the identity of genetic and environmental modifiers that determine phenotypic heterogeneity—that define the frontier of current DADA2 research. This review critically evaluates both established knowledge and persistent uncertainties, positioning DADA2 as an instructive model for the study of monogenic immune vasculopathy.

## 1. Introduction

### 1.1. The Emergence of Monogenic Vasculitis

The systemic vasculitides comprise a heterogeneous group of disorders characterized by inflammation of blood vessel walls, leading to tissue ischemia, organ dysfunction, and significant morbidity. For decades, the pathogenesis of vasculitis remained poorly understood, with classification based primarily on vessel size, histopathology, and clinical phenotype rather than underlying etiology [1]. The discovery of monogenic forms of vasculitis has fundamentally transformed this landscape, demonstrating that a single genetic defect can recapitulate the entire spectrum of vascular inflammation observed in complex polygenic diseases [2]. Among these monogenic vasculitides, DADA2 stands as the most well-characterized and instructive paradigm.

Before 2014, the concept of a monogenic vasculitis was largely confined to rare entities such as adenosine deaminase 1 (*ADA1*) deficiency, which presents with severe combined immunodeficiency and systemic inflammation, and a handful of autoinflammatory syndromes with vascular involvement. The identification of DADA2 as a genetic cause of early-onset stroke and vasculopathy opened a new chapter in vascular immunology [3,4]. DADA2 is now recognized as the most common monogenic mimic of polyarteritis nodosa (PAN), accounting for a substantial proportion of childhood-onset PAN and an unknown fraction of adult-onset cases [5]. Importantly, DADA2 has also expanded our understanding of the non-redundant roles of adenosine deaminase 2 in immune homeostasis, endothelial function, and hematopoiesis.

Positioning DADA2 within the nosology of vasculitis has important implications. Unlike polygenic vasculitides such as granulomatosis with polyangiitis or microscopic polyangiitis, DADA2 exemplifies a streamlined pathophysiology: a single enzymatic deficiency triggers a cascade of immune dysregulation culminating in vascular inflammation [6]. This genetic clarity provides a unique opportunity to trace the molecular steps from genotype to vascular phenotype, offering insights that may be applicable to the broader spectrum of inflammatory vascular diseases.

### 1.2. Discovery of DADA2

In 2014, two landmark papers simultaneously identified biallelic mutations in *ADA2* (formerly *CECR1*) as the cause of an autosomal recessive syndrome of early-onset stroke, fever, livedo racemosa, and vasculopathy. Zhou et al. described patients with childhood-onset ischemic strokes and a PAN-like vasculopathy, while Navon Elkan et al. independently reported similar findings in patients with a familial form of PAN [3,4]. Both studies leveraged complementary approaches: Zhou et al. used whole-exome sequencing in a cohort of patients with unexplained early-onset stroke and vascular inflammation, while Navon Elkan et al. employed linkage analysis in consanguineous families with PAN-like disease.

The discovery rapidly catalyzed international efforts to characterize the clinical spectrum and pathophysiology of DADA2. Within the first year, multiple case series confirmed the triad of vasculitis, stroke, and hematologic abnormalities, and established the efficacy of TNF inhibition [7,8]. Subsequent cohorts expanded the phenotypic spectrum to include immunodeficiency, autoimmune cytopenias, lymphoproliferation, and liver involvement [9,10]. To date, over 400 patients have been reported worldwide, with the true prevalence likely higher due to underdiagnosis in both pediatric and adult populations [11]. The availability of commercial genetic testing and increased awareness among rheumatologists, hematologists, immunologists, and neurologists continues to accelerate case identification.

Within two years of the initial description of DADA2, the first clinical trials of anti-TNF therapy were underway, and HSCT outcomes have since been systematically evaluated in multi-center registries [12,13]. This accelerated timeline reflects both the urgent need for effective therapies in a devastating childhood-onset disease and the straightforward biological rationale for targeted intervention. DADA2 has thus become a model for how single-gene discovery can rapidly translate into precision therapeutic strategies.

**Figure 1:**
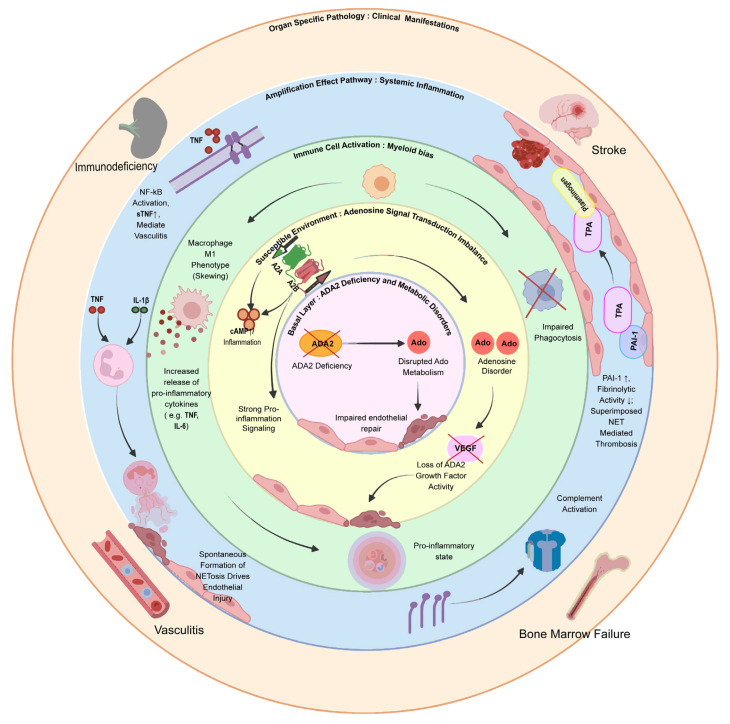
Hierarchical model of ADA2 deficiency linking molecular dysfunction to clinical phenotypes.

In this review, we synthesize current advances in DADA2 research from molecular biology and immunopathogenesis to clinical heterogeneity, multi-omics discoveries, and emerging precision therapies. As an overview of the disease framework discussed throughout this article, Figure 1 summarizes the major biological functions of ADA2, the key pathogenic mechanisms triggered by ADA2 deficiency, and the principal clinical phenotypes that define DADA2 (Figure 1).

## 2. ADA2 Biology

### 2.1. Molecular Structure and Enzymatic Function

Adenosine deaminase 2 (ADA2) is encoded by the *ADA2* gene located on chromosome 22q11.1, within the region commonly deleted in DiGeorge syndrome [14,15]. The protein is a 511-amino-acid secreted enzyme that exists as a homodimer. ADA2 shares approximately 20% sequence identity with ADA1 but possesses distinct structural and functional features. The enzyme comprises a signal peptide, an immunoglobulin-like (Ig) domain, and a catalytic domain containing the characteristic α/β-TIM barrel fold of the adenosine deaminase family [16]. Unlike ADA1, which is a cytoplasmic enzyme with high catalytic efficiency for adenosine, ADA2 is secreted and has a lower catalytic rate but broader substrate specificity, including activity toward both adenosine and 2′-deoxyadenosine [16].

The enzymatic function of ADA2 is to catalyze the irreversible deamination of adenosine to inosine and 2′-deoxyadenosine to 2′-deoxyinosine, thereby regulating extracellular adenosine levels. This reaction is critical because adenosine exerts pleiotropic effects through four G-protein-coupled receptors (A1, A2A, A2B, A3), influencing inflammation, vascular tone, platelet aggregation, and immune cell function [17]. Under normal conditions, extracellular adenosine levels are tightly controlled by the coordinated action of ADA2 and other ectonucleotidases. In DADA2, the loss of ADA2 enzymatic activity leads to elevated extracellular adenosine, which can paradoxically promote both pro-inflammatory and anti-inflammatory effects depending on the receptor subtype and cellular context [18].

The pH optimum of ADA2 (pH 5.5–6.5) differs markedly from that of ADA1 (pH 7.0–8.0), suggesting that ADA2 is primarily active in the acidic microenvironment of inflamed or hypoxic tissues [16]. This pH-dependent activity profile positions ADA2 as a stress-responsive enzyme that modulates adenosine signaling specifically at sites of inflammation. The clinical observation that DADA2 patients develop disease manifestations predominantly in the vascular and hematopoietic compartments underscores the tissue-specific importance of this pathway.

### 2.2. Macrophage Biology and ADA2 Secretion

ADA2 is predominantly produced by myeloid cells, particularly monocytes and macrophages, with minimal expression in other cell types [19]. The differentiation of monocytes into macrophages, especially toward the M2 (anti-inflammatory) polarization state, is associated with increased ADA2 secretion. Conversely, M1 (pro-inflammatory) macrophages express lower levels of ADA2 [20]. This lineage-specific expression pattern suggests that ADA2 is an integral component of the macrophage functional program, linking cellular metabolism to immune modulation.

Studies of ADA2-deficient macrophages have revealed profound functional abnormalities. In the absence of ADA2, macrophages exhibit altered polarization dynamics, with a skew toward a pro-inflammatory M1-like phenotype characterized by increased production of TNF, and reduced expression of M2-associated markers [21]. This polarization defect is accompanied by metabolic reprogramming, including increased glycolysis and reduced oxidative phosphorylation, a pattern reminiscent of the classical Warburg effect observed in activated immune cells [22]. The mechanistic link between ADA2 deficiency and macrophage polarization involves adenosine receptor signaling: elevated extracellular adenosine in ADA2-deficient conditions preferentially engages the A2B receptor, which has been shown to promote pro-inflammatory cytokine production in macrophages [21,23].

Furthermore, ADA2-deficient macrophages display impaired efferocytosis—the clearance of apoptotic cells—a function critical for resolving inflammation and maintaining tissue homeostasis [24]. This defect may contribute to the accumulation of cellular debris and secondary necrosis at sites of vascular injury, perpetuating a cycle of inflammation and tissue damage. The combination of excessive TNF production, impaired debris clearance, and altered metabolic programming positions the macrophage as a central orchestrator of the DADA2 inflammatory phenotype.

### 2.3. Endothelial Homeostasis

Beyond its enzymatic function, ADA2 possesses a well-documented but incompletely understood role as a growth factor for endothelial cells. Zavialov et al. first demonstrated that ADA2 promotes endothelial cell proliferation and tube formation in vitro, an activity that appears to be independent of its adenosine deaminase catalytic activity [25]. The mechanism is thought to involve engagement of cell surface proteoglycans and integrin receptors, leading to activation of the ERK1/2 and PI3K/Akt signaling pathways [26]. This growth factor activity distinguishes ADA2 from ADA1 and explains why *ADA1* deficiency does not recapitulate the vascular phenotype of DADA2. However, a fundamental unresolved question is whether the vasculopathy of DADA2 is driven primarily by the loss of this growth factor activity (i.e., impaired endothelial repair) or by the enzymatic defect (i.e., adenosine dysregulation leading to inflammation) [27]. We hypothesize that these two functions are not mutually exclusive but rather converge to create a ‘double-hit’ vulnerability, in which ADA2 deficiency simultaneously impairs vascular repair mechanisms and unleashes inflammatory pathways that damage the vessel wall. Disentangling their relative contributions remains a priority for understanding disease pathogenesis.

Endothelial cells derived from DADA2 patients exhibit reduced proliferative capacity, increased sensitivity to inflammatory stimuli, and impaired barrier function [28]. In vitro, ADA2-deficient endothelial cells show increased expression of adhesion molecules (VCAM-1, ICAM-1) following TNF stimulation, as well as reduced expression of tight junction proteins (ZO-1, occludin) [3]. These abnormalities likely contribute to the increased vascular permeability, leukocyte infiltration, and vessel wall inflammation observed in DADA2 patients. These findings further support the ‘double-hit’ model, in which the disruption of endothelial repair mechanisms and the activation of pro-inflammatory pathways act in concert to drive the onset and progression of vascular damage.

Adenosine metabolism plays a crucial role in the endothelial niche. Endothelial cells express high levels of adenosine receptors, and adenosine signaling modulates vascular tone, angiogenesis, and endothelial barrier function [29,30]. In DADA2, the disruption of adenosine homeostasis—with elevated extracellular adenosine in some contexts and deficiency in others—creates a complex microenvironmental dysregulation. This metabolic disturbance, combined with the loss of ADA2 growth factor activity, renders the endothelium susceptible to inflammatory injury and impairs its capacity for repair following damage [31].

### 2.4. Adenosine Metabolism and Immune Modulation

The adenosine signaling pathway is a critical checkpoint in immune regulation. Under physiological conditions, extracellular adenosine levels rise at sites of tissue damage and inflammation, serving as a negative feedback signal that limits the magnitude and duration of immune responses [32]. This immunoregulatory function is primarily mediated through the A2A receptor, which couples to Gαs and increases intracellular cAMP, thereby suppressing the activation of T cells, neutrophils, and macrophages [32]. Whether the chronic adenosine elevation in DADA2 paradoxically desensitizes A2A receptor signaling—thereby converting a normally anti-inflammatory signal into a pro-inflammatory state through preferential engagement of low-affinity A2B receptors [33]—remains an unresolved question. If confirmed, adenosine receptor-modulating agents could represent a more proximal therapeutic strategy.

In DADA2, the loss of ADA2 creates a paradox: while total adenosine deaminase activity in the blood is reduced, the relative contribution of ADA1 versus ADA2 varies by tissue compartment. In the extracellular milieu of inflamed tissues, where ADA2 is normally active, its deficiency leads to local adenosine accumulation. This accumulation may selectively activate A2B receptors, which differ from A2A in their lower affinity for adenosine and their ability to signal through Gαq in addition to Gαs [34]. A2B receptor activation in macrophages has been linked to increased IL-6 and TNF production, providing a mechanistic link between ADA2 deficiency and the inflammatory phenotype [21]. Conversely, in the bone marrow microenvironment, altered adenosine signaling disrupts hematopoietic stem and progenitor cell homeostasis, potentially contributing to the hematologic manifestations of DADA2 [35].

The purine metabolism network in DADA2 is further complicated by the fact that ADA2 is a major source of extracellular adenosine deaminase activity in the serum. Patients with DADA2 have markedly reduced serum adenosine deaminase activity, a finding that is exploited as a diagnostic screening tool [36]. However, intracellular ADA1 activity is preserved, meaning that the metabolic consequences of ADA2 deficiency are largely confined to the extracellular compartment. This compartment-specific defect explains the unique clinical phenotype of DADA2, which differs substantially from ADA1 deficiency in its absence of severe lymphopenia and accumulation of intracellular deoxyadenosine metabolites [37]. Understanding these metabolic nuances has informed therapeutic strategies that target downstream inflammatory pathways rather than attempting to replace the missing enzyme.

## 3. Immunopathogenesis

### 3.1. TNF Axis

The TNF (tumor necrosis factor) axis occupies center stage in DADA2 immunopathogenesis. Functional studies revealed that monocytes and macrophages from DADA2 patients produce excessive amounts of TNF both at baseline and upon stimulation [24]. This finding was consistent across multiple patient cohorts and provided the rationale for anti-TNF therapy, which remains the most effective treatment for the vasculitic manifestations of the disease [8]. However, a critical unresolved question is whether TNF overproduction represents the primary pathogenic driver or rather an amplifier of a more fundamental defect in ADA2-deficient myeloid cells. This distinction has important therapeutic implications: if TNF is primarily an amplifier, upstream targets—such as A2B receptor signaling or metabolic reprogramming—may need to be addressed for complete disease control [23].

The mechanisms driving TNF overproduction in DADA2 are multifactorial. At the transcriptional level, ADA2 deficiency leads to increased NF-κB activation in myeloid cells, resulting in enhanced transcription of *TNF* and other *NF-κB* target genes [33]. This is accompanied by altered post-translational regulation, including increased TNF-converting enzyme (TACE/ADAM17) activity, which promotes the shedding of soluble TNF from the cell surface [38]. The resulting imbalance between membrane-bound and soluble TNF is thought to contribute to the distinctive vasculitic phenotype, as soluble TNF has enhanced bioavailability for endothelial activation compared with its membrane-bound counterpart.

#### TNF Receptor Signaling

TNF exerts its effects through two receptors: TNFR1 (p55), which is broadly expressed and mediates pro-inflammatory and apoptotic signaling, and TNFR2 (p75), which is primarily expressed on immune cells and endothelial cells and can promote cell survival and proliferation [39]. In DADA2, the TNF-rich microenvironment leads to sustained TNFR1 signaling in endothelial cells, triggering the expression of adhesion molecules, chemokines, and pro-coagulant factors that collectively promote vascular inflammation and thrombosis. TNFR2 signaling, in contrast, may have protective effects in some contexts, and the differential engagement of these two receptors has been proposed to influence the clinical phenotype [40].

### 3.2. IFN Axis

Beyond TNF, the interferon (IFN) axis contributes to the inflammatory milieu in DADA2, though its role appears to be more nuanced and patient-dependent [41]. Transcriptomic analyses of peripheral blood mononuclear cells from DADA2 patients have revealed a type I interferon signature in a subset of patients, with upregulation of IFN-stimulated genes (ISGs) including *IFIT1*, *IFIT3*, *MX1*, and *OAS1* [6]. This IFN signature correlates with the presence of anti-nuclear antibodies and lymphoproliferation, suggesting a link between IFN dysregulation and the autoimmune features of DADA2 [42].

The activation of the IFN pathway in DADA2 has been proposed to be secondary to TNF-driven inflammation or may alternatively reflect a parallel pathway triggered by the accumulation of endogenous nucleic acid species due to impaired clearance of apoptotic debris. Consistent with the latter hypothesis, ADA2-deficient macrophages show reduced expression of *DNase II* and *TREX1*, enzymes involved in the clearance of cytosolic DNA that, when deficient, lead to type I IFN production via the *cGAS*-*STING* pathway [28]. This suggests a mechanistic convergence between DADA2 and the interferonopathies, a group of monogenic diseases characterized by constitutive IFN signaling.

The clinical significance of the IFN signature in DADA2 remains an active area of investigation. Unlike TNF-driven manifestations, which respond uniformly to anti-TNF therapy, IFN-related features such as lymphoproliferation and autoantibody production may persist despite TNF blockade [43,44,45]. An unresolved but clinically important question is whether the IFN signature is a primary consequence of ADA2 deficiency (perhaps through impaired clearance of endogenous nucleic acids by ADA2-deficient macrophages [46]) or a secondary phenomenon driven by TNF-induced tissue damage and the release of damage-associated molecular patterns. Some DADA2 patients display a prominent IFN signature, whereas others do not—raising the question of what determines this divergence. Genetic variation in IFN pathway genes may contribute to this heterogeneity. Alternatively, stochastic differences in the acquisition of endogenous nucleic acid species could shape individual IFN responses. Whether adjunctive therapies targeting the IFN pathway (e.g., JAK inhibitors) will benefit these patients remains an open question, with case reports suggesting potential efficacy in refractory cases [47], but larger prospective studies are needed to define the optimal therapeutic strategy.

### 3.3. Neutrophils and NETosis

Neutrophils have emerged as key effector cells in DADA2 vasculopathy. Peripheral blood neutrophils from DADA2 patients display enhanced spontaneous NETosis—the release of neutrophil extracellular traps composed of DNA, histones, and granular enzymes [33]. NETosis is a double-edged sword in vascular inflammation: while NETs are important for host defense against pathogens, their excessive formation can damage the endothelium, expose immunostimulatory molecules, and promote thrombosis [48].

The propensity for NETosis in DADA2 appears to be driven by the inflammatory cytokine milieu, particularly by TNF and IL-1β, both of which are elevated in DADA2 patients. In vitro, treatment of healthy neutrophils with DADA2 patient serum stimulates NET formation, an effect that is abrogated by TNF neutralization. Furthermore, ADA2-deficient neutrophils themselves may be intrinsically primed for NETosis, as ADA2 has been shown to regulate neutrophil activation through adenosine receptor signaling [49].

The consequences of excessive NETosis in DADA2 are manifold. NETs directly injure endothelial cells, promote the exposure of von Willebrand factor (vWF) and tissue factor, and provide a scaffold for platelet adhesion and thrombus formation [50]. In the context of DADA2 vasculitis, NETs have been visualized in the inflamed vessel walls of affected tissues, co-localizing with areas of endothelial disruption and leukocyte infiltration. NETs also contain proteases such as neutrophil elastase and matrix metalloproteinases that degrade the vascular extracellular matrix, contributing to vessel wall damage and aneurysm formation [51]. The contribution of NETosis to the thrombotic tendency in DADA2, particularly the high risk of ischemic stroke, represents a critical link between the inflammatory and thrombotic manifestations of the disease.

### 3.4. Complement and Coagulation Crosstalk

Emerging evidence suggests interplay between the complement system and coagulation pathways in DADA2 vasculopathy. Complement activation products, including C3a and C5a, are elevated in DADA2 patient plasma and correlate with disease activity. These anaphylatoxins exert chemotactic effects on neutrophils and monocytes, promote endothelial activation, and can trigger NETosis. The complement system may also interact with the coagulation cascade, as NETs provide a surface for complement factor deposition, and complement activation products can induce tissue factor expression on endothelial cells, promoting a procoagulant state [52].

The prothrombotic state in DADA2 is further characterized by elevated levels of plasminogen activator inhibitor-1 (PAI-1) and reduced fibrinolytic activity [53]. The combination of endothelial activation, NET-mediated thrombosis, complement dysregulation, and impaired fibrinolysis creates a perilous milieu that explains the high incidence of ischemic stroke and other thrombotic events in DADA2—events that often precede the diagnosis by years.

### 3.5. Vasculopathy: Endothelial Injury Mechanisms

The vascular pathology in DADA2 represents the convergence of multiple pathogenic pathways on the vessel wall. Histopathological examination of affected arteries reveals a transmural inflammatory infiltrate composed of neutrophils, macrophages, and T lymphocytes, accompanied by fibrinoid necrosis of the media and intimal hyperplasia [28]. These findings are indistinguishable from classic PAN, underscoring DADA2 as a monogenic phenocopy of this polygenic disease.

Endothelial injury in DADA2 results from the combined effects of: (1) direct cytotoxicity from TNF and other inflammatory cytokines; (2) NET-mediated endothelial damage; (3) reduced endothelial repair capacity due to the loss of ADA2 growth factor activity; (4) complement-mediated injury; and (5) altered adenosine signaling affecting endothelial barrier function [4,21,23,54]. Each of these mechanisms alone may be insufficient to cause significant vascular pathology, but their convergence in DADA2 produces catastrophic vessel wall damage. The predilection for cerebral vessels, particularly the internal carotid artery and middle cerebral artery territories, reflects the unique hemodynamic and metabolic properties of the cerebral circulation, though the precise determinants of this anatomical selectivity remain incompletely understood.

Intriguingly, not all DADA2 patients develop clinically apparent vasculitis. Some present primarily with hematologic manifestations (pure red cell aplasia, pancytopenia) or immunodeficiency without evidence of vascular inflammation [55]. This phenotypic divergence suggests the existence of modifying factors—genetic, epigenetic, or environmental—that determine the tissue-specific expression of the disease. Understanding these modifiers is a major priority for predicting disease course and personalizing therapeutic strategies.

Proposed hierarchical model. On the basis of the findings presented in this section, we propose a working hierarchical model of DADA2 immunopathogenesis (Figure 1). At the base (Layer 1), ADA2 deficiency disrupts extracellular adenosine metabolism and impairs endothelial repair. This creates a permissive environment (Layer 2) in which adenosine dysregulation—characterized by A2A receptor desensitization and A2B receptor predominance—converts a normally anti-inflammatory signal into a pro-inflammatory state, while the loss of ADA2 growth factor activity compromises endothelial barrier integrity. The resultant adenosine-driven inflammation triggers myeloid activation (Layer 3), with macrophage skewing toward a pro-inflammatory M1 phenotype, enhanced TNF production, and impaired efferocytosis. These myeloid abnormalities amplify downstream effector pathways (Layer 4), including TNF-mediated vascular inflammation, NETosis-driven endothelial injury, complement activation, and a prothrombotic state. The convergence of these amplified pathways on the vessel wall produces organ-specific pathology (Layer 5), with the particular clinical manifestations—vasculitis, stroke, bone marrow failure, or immunodeficiency—determined by the tissue-specific expression of these hierarchical layers. This model provides a mechanistic framework for understanding both the shared inflammatory pathology and the phenotypic divergence observed across DADA2 patients.

## 4. Clinical Heterogeneity

### 4.1. Vasculitis Phenotype

The vasculitic manifestations of DADA2 are the most characteristic and frequently recognized presenting features. DADA2-associated vasculitis closely resembles PAN, affecting medium-sized arteries with a predilection for the skin, nervous system, gastrointestinal tract, and kidneys [4]. Cutaneous findings are nearly universal and include livedo racemosa (a striking purplish, reticular rash that persists upon warming, distinguishing it from livedo reticularis), subcutaneous nodules, digital ischemia, and necrotic ulcers [56]. These skin findings often provide the first clinical clue to the diagnosis.

Gastrointestinal involvement presents with abdominal pain, gastrointestinal bleeding, and—in severe cases—intestinal perforation due to mesenteric ischemia [57]. Renal artery involvement can lead to hypertension and renal impairment, though frank renal failure is less common than in classic PAN. Testicular pain and tenderness, reflecting orchitis due to involvement of testicular arteries, is a characteristic but underrecognized feature [58]. The vasculitic manifestations are often relapsing-remitting, with flares triggered by infections, vaccinations, or other immune stimuli. Importantly, the vasculitis of DADA2 typically responds rapidly to anti-TNF therapy, with most patients achieving complete or near-complete remission within weeks of treatment initiation [6].

### 4.2. Stroke and Neurological Involvement

Stroke is the most devastating manifestation of DADA2 and the presenting feature in approximately 30–50% of pediatric cases [3,59,60]. DADA2-associated strokes are typically ischemic, lacunar in appearance, and involve the deep brain structures (basal ganglia, thalamus, internal capsule) and the brainstem. The median age at first stroke is 4–6 years, though strokes have been reported in infancy and in adults [8,61]. Recurrent strokes are common in untreated patients, leading to cumulative neurological injury and cognitive impairment.

The pathogenesis of stroke in DADA2 involves both vasculitic and thrombotic mechanisms. Inflammatory infiltration of the cerebral vessel wall leads to stenosis, occlusion, and impaired autoregulation of cerebral blood flow. Superimposed thrombosis, promoted by NETosis and endothelial activation, converts a hemodynamically compromised vessel into a completely occluded one [62]. Neuroimaging typically reveals multifocal, chronic ischemic changes with associated cerebral atrophy. Magnetic resonance angiography may demonstrate stenosis or occlusion of the internal carotid, middle cerebral, and basilar arteries, sometimes accompanied by the development of collateral circulation reminiscent of moyamoya disease [63,64]. One of the most intriguing yet poorly understood features of DADA2 is the striking cerebrovascular tropism of the vasculitic process. This predilection has been proposed to reflect the unique metabolic demands of the cerebral endothelium, which has a particularly high dependence on ADA2-mediated endothelial growth factor signaling [63], or the sensitivity of the cerebral circulation to adenosine-mediated vasoregulatory disturbances [65]. Understanding the mechanisms underlying this tissue selectivity could reveal fundamental principles of organ-specific vascular vulnerability.

Beyond overt stroke, DADA2 patients may experience subclinical ischemic events, cognitive decline, headaches, and cranial nerve palsies. The burden of silent cerebral ischemia, as detected by magnetic resonance imaging, is increasingly recognized as a marker of ongoing disease activity and a potential indication for treatment intensification [66]. Prevention of stroke—whether through anti-TNF therapy or HSCT—is the primary therapeutic goal in the management of DADA2.

### 4.3. Hematologic Phenotype

The hematologic manifestations of DADA2 are protean and can mimic primary bone marrow failure syndromes. Pure red cell aplasia (PRCA) is a well-recognized complication, presenting in some patients as the dominant or sole clinical feature [43]. Pancytopenia, with variable degrees of anemia, thrombocytopenia, and neutropenia, is also common and may evolve over the disease course. Bone marrow examination often reveals hypocellularity with dyserythropoiesis, though cellularity can range from profoundly hypocellular to normocellular [35].

The mechanisms underlying bone marrow failure in DADA2 are incompletely understood but likely involve both intrinsic hematopoietic stem cell (HSC) defects and extrinsic suppression by the inflammatory microenvironment. ADA2 is expressed in the bone marrow niche, and its deficiency may impair HSC maintenance and differentiation [35]. Additionally, elevated TNF and IFN levels in the bone marrow microenvironment exert suppressive effects on hematopoiesis, contributing to the cytopenias [67]. As discussed in above, the limited responsiveness of hematologic manifestations to TNF blockade stems from TNF-independent pathogenic mechanisms in the bone marrow—including T-cell-mediated suppression, altered adenosine signaling in the HSC niche, and IFN-driven myelosuppression [12,67]—pathways that are not adequately targeted by TNF inhibitors alone. This distinction has important therapeutic implications: patients with hematologic-predominant DADA2 may benefit from earlier consideration of HSCT or alternative targeted therapies rather than prolonged TNFi monotherapy.

DADA2 also presents with features of immune dysregulation that extend beyond the bone marrow. Autoimmune hemolytic anemia, immune thrombocytopenia, and Evans syndrome have been reported [68]. Lymphoproliferation, manifesting as splenomegaly, lymphadenopathy, and even lymphoma, is increasingly recognized as a component of the DADA2 spectrum [55]. These features highlight the dual nature of DADA2 as both an autoinflammatory disease (driven by innate immune dysregulation) and an autoimmune disease (with adaptive immune system involvement).

### 4.4. Immunodeficiency

Humoral immunodeficiency is a frequent but often overlooked component of DADA2. Common variable immunodeficiency (CVID)-like features, including hypogammaglobulinemia, specific antibody deficiency, and impaired B cell differentiation, have been documented in up to 30% of patients [69,70]. The severity of immunodeficiency is highly variable, ranging from subclinical antibody abnormalities to clinically significant infections requiring immunoglobulin replacement therapy.

The B cell defect in DADA2 appears to be intrinsic and related to the role of ADA2 in B cell development and function. Patients often exhibit reduced class-switched memory B cells, impaired in vitro immunoglobulin production, and diminished responses to vaccination [71]. T cell numbers and function are typically preserved, though some patients show reduced T cell proliferative responses and skewing of the T cell receptor repertoire [71]. The combination of autoinflammation, autoimmunity, and immunodeficiency in DADA2 places it squarely within the expanding category of diseases at the intersection of these traditionally distinct diagnostic categories, exemplified by the “immune dysregulation” disease framework.

Clinical management of immunodeficiency in DADA2 requires a tailored approach. Immunoglobulin replacement therapy is indicated for patients with recurrent infections and demonstrated antibody deficiency. However, the decision to treat with immunosuppressive therapies (TNF inhibitors, corticosteroids) must be weighed against the risk of exacerbating the underlying immunodeficiency. For patients with severe hematologic disease and immunodeficiency, HSCT offers the potential for definitive correction of both the inflammatory and immune defects [11,55]. The major clinical phenotypes of DADA2 are systematically summarized in Table 1, which outlines the core clinical features, typical onset characteristics, key laboratory and imaging findings, and first-line therapeutic strategies for each phenotypic category.

## 5. Genotype–Phenotype Correlation

One of the most intriguing aspects of DADA2 is the complex relationship between specific ADA2 mutations and the resulting clinical phenotype. Over 100 distinct pathogenic ADA2 mutations have been described, distributed throughout the coding region and including missense, nonsense, frameshift, splice-site, and deletion variants [6]. Several broad genotype–phenotype correlations have emerged, though significant inter- and intra-familial variability complicates prediction.

### 5.1. Broad Genotype–Phenotype Correlations

Mutations that result in complete loss of ADA2 protein expression or enzyme activity (nonsense, frameshift, large deletions) are generally associated with more severe and multi-system disease, particularly with hematologic involvement [46,72]. Patients with biallelic null mutations tend to present early in life with severe vasculitis, stroke, and bone marrow failure. In contrast, missense mutations that partially preserve enzyme activity or protein expression are more often associated with a vasculitic-predominant phenotype, later disease onset, and milder hematologic manifestations [43].

The p.Gly47Arg (G47R) variant, one of the most common pathogenic mutations, provides a illustrative example. Homozygosity for G47R is associated with a predominantly vasculitic phenotype, with a high penetrance of stroke and cutaneous vasculitis but relatively spared hematologic function [73,74]. In contrast, compound heterozygosity for G47R with a null allele results in more severe disease, often including hematologic involvement. The p.Gly358Arg (G358R) variant, also relatively common in certain populations, has been associated with pure red cell aplasia when present in the homozygous state, whereas compound heterozygotes display more variable phenotypes [75].

### 5.2. Limitations and Modifying Factors

Despite these emerging correlations, the genotype–phenotype relationship in DADA2 is far from deterministic. Significant phenotypic variability is observed among patients carrying identical mutations, including affected siblings within the same family [6,76]. This variability implicates the contribution of modifier genes, epigenetic factors, environmental triggers (e.g., infections), and stochastic developmental effects in shaping disease expression. The identification of these modifying factors represents a major frontier in DADA2 research that may yield insights applicable to human genetic disease more broadly. Emerging evidence suggests that at least three layers of modification operate: (1) genetic variation in TNF, IFN, and adenosine pathway genes that modulate the downstream consequences of ADA2 deficiency; (2) epigenetic reprogramming of myeloid progenitors driven by chronic inflammation (trained immunity); and (3) environmental factors—particularly the timing and nature of infectious exposures—that trigger disease onset or flares. An unresolved but clinically important question is whether these modifiers can be systematically identified through large-scale genome-wide association studies in DADA2 cohorts, or whether the rarity of the disease will necessitate more creative approaches such as CRISPR screens in patient-derived iPSC models.

From a clinical standpoint, the limitations of genotype–phenotype prediction underscore the importance of comprehensive clinical phenotyping in guiding therapeutic decisions. While the presence of certain mutations (e.g., biallelic null mutations) may argue for early consideration of HSCT, the treatment of any individual patient must be based on the full clinical picture rather than genetic data alone [77]. Future studies integrating genomics with transcriptomics, proteomics, and clinical data will be essential for refining risk stratification and personalizing treatment approaches.

### 5.3. Diagnostic Approach and Biomarkers

The diagnostic approach to DADA2 begins with clinical suspicion in patients presenting with early-onset stroke, unexplained vasculitis resembling polyarteritis nodosa, hematologic abnormalities of unclear etiology, or humoral immunodeficiency with vascular features. First-line laboratory evaluation consists of measurement of serum ADA2 enzymatic activity, which is markedly reduced in affected individuals and serves as a rapid and cost-effective screening tool [36]. However, a normal ADA2 activity level does not entirely exclude the diagnosis, particularly in patients with hypomorphic missense mutations that retain partial enzymatic function, and confirmatory genetic testing is therefore recommended in all suspected cases.

Genetic testing typically involves sequencing of the *ADA2* gene (formerly *CECR1*), including coding exons and flanking intronic regions, with deletion/duplication analysis by multiplex ligation-dependent probe amplification (MLPA) or chromosomal microarray to detect large deletions or rearrangements that may be missed by sequencing alone [76]. The identification of biallelic pathogenic ADA2 mutations confirms the diagnosis. Given the broad phenotypic spectrum and the availability of specific therapy, early genetic testing is warranted in patients with suggestive clinical features, as diagnostic delay is associated with accumulative morbidity, particularly stroke recurrence [59].

## 6. Multi-Omics and Single-Cell Insights

The advent of high-resolution omics technologies has begun to illuminate the cellular and molecular complexity underlying DADA2 at unprecedented resolution. Single-cell RNA sequencing (scRNA-seq) studies of peripheral blood and affected tissues from DADA2 patients have revealed distinct alterations in the myeloid compartment, including expansion of a pro-inflammatory monocyte subset characterized by high expression of S100A8/S100A9 (calprotectin), IL1B, and TNF [78]. This monocyte subpopulation shows transcriptional signatures consistent with trained immunity, suggesting that ADA2 deficiency induces lasting epigenetic reprogramming of myeloid progenitors.

### 6.1. Single-Cell Transcriptomics

Myeloid trajectory analysis has identified a skewing of monocyte-to-macrophage differentiation toward an inflammatory intermediate state with reduced capacity for terminal M2 polarization [24]. This differentiation block appears to be driven by autocrine TNF signaling and can be partially reversed by anti-TNF therapy. Notably, the myeloid transcriptomic signature is more pronounced in patients with active vasculitis compared with those in remission, suggesting its potential utility as a biomarker of disease activity [79].

Spatial transcriptomic analysis of skin and arterial biopsy specimens has begun to characterize the tissue microenvironment in DADA2 vasculitis. These studies have revealed that affected vessels contain spatially organized immune niches comprising macrophages, CD8+ T cells, neutrophils, and fibroblasts expressing extracellular matrix remodeling enzymes [42]. The perivascular niche in DADA2 is characterized by a type I interferon signature and heightened expression of chemokines such as CXCL9, CXCL10, and CXCL11 that recruit activated T cells and monocytes to the vessel wall. These spatial findings provide a structural framework for understanding how systemic ADA2 deficiency leads to focal vascular pathology.

### 6.2. Cytokine Profiling and Multi-Omics Integration

Cytokine profiling studies have consistently shown elevations of TNF, IL-6, IL-1β, IL-18, and IP-10 (CXCL10) in DADA2 patient plasma, with the TNF/IL-18 ratio potentially distinguishing vasculitic from hematologic-predominant subtypes [80]. Proteomic analyses have further identified signatures of endothelial activation (elevated vWF, soluble VCAM-1, angiopoietin-2), neutrophil degranulation (increased myeloperoxidase, neutrophil elastase), and complement activation (elevated C5a, sC5b-9) [33,42]. Integration of these multi-omics datasets using machine learning approaches is beginning to identify composite biomarkers that outperform any single analyte for diagnosis, disease activity monitoring, and prediction of treatment response [81].

The concept of DADA2 as a disease of disturbed “immune-endothelial crosstalk” has been reinforced by single-cell studies examining the endothelial compartment. Endothelial cells from DADA2 patients show transcriptional evidence of activation (E-selectin, VCAM1 upregulation) and reduced expression of genes involved in vascular repair (VEGFA, ANGPT1). These abnormalities are most pronounced in the cerebral microvasculature, consistent with the clinical predilection for neurological involvement. Single-cell studies have also identified a population of “activated” endothelial cells that express MHC class II molecules and may function as antigen-presenting cells, potentially contributing to local T cell activation within the vessel wall [78]. This observation highlights unanswered questions about the role of adaptive immunity in DADA2 vasculitis, a topic that has received less attention than the innate immune contributions.

### 6.3. Senescence-Associated Programs and Aging-Related Mechanisms

Recent studies have begun to reveal unexpected links between ADA2 deficiency and biological pathways commonly associated with aging [22,71]. Although DADA2 is not traditionally classified as a premature aging syndrome, emerging evidence suggests that chronic ADA2 deficiency may engage cellular programs related to senescence, stem cell exhaustion, and tissue aging [82,83].

One of the most intriguing observations comes from the bone marrow microenvironment. Mesenchymal stromal cells (MSCs) derived from DADA2 patients exhibit a constellation of aging-like phenotypes, including impaired proliferative capacity, accumulation of DNA damage, and induction of senescence-associated secretory phenotype (SASP). The concept of inflammaging—the reciprocal reinforcement between chronic inflammation and cellular senescence—provides a useful framework for these findings, though whether inflammation–senescence crosstalk directly contributes to disease progression in DADA2 remains unknown. Beyond the hematopoietic compartment, hallmarks of DADA2 including endothelial dysfunction and impaired tissue repair overlap conceptually with biological processes implicated in vascular aging, yet direct evidence linking ADA2 deficiency to accelerated biological aging remains limited.

Future studies integrating single-cell transcriptomics, epigenomic profiling, and longitudinal analyses of patient-derived stem cell populations will be necessary to determine whether senescence represents a secondary consequence of chronic inflammation or a fundamental component of DADA2 pathophysiology. Clarifying this relationship may not only improve understanding of hematologic manifestations in DADA2 but also provide broader insights into the interplay between chronic inflammation, stem cell dysfunction, and aging-associated disease [13].

## 7. Precision Therapeutics

### 7.1. TNF Inhibition

Anti-TNF therapy is the cornerstone of DADA2 management and was one of the earliest examples of rational, mechanism-based therapy for a monogenic vasculitis. The rationale is straightforward: excessive TNF production is a cardinal feature of the immunopathogenesis, and neutralization of this cytokine should interrupt the inflammatory cascade leading to vascular injury. Clinical experience has borne out this prediction with remarkable consistency.

The largest published cohort of DADA2 patients treated with TNF inhibitors (TNFi) demonstrated that etanercept, infliximab, and adalimumab are all highly effective in preventing stroke, resolving fevers, normalizing inflammatory markers, and healing cutaneous vasculitic lesions. In one multicenter study of over 100 patients, TNFi therapy reduced the annual incidence of stroke from approximately 0.4 events per patient-year to zero. The response to TNFi is typically rapid, with clinical improvement evident within days to weeks of treatment initiation.

The choice among TNFi agents is guided by practical considerations. Etanercept, a soluble TNFR2-Fc fusion protein, has been the most widely used agent in DADA2, likely due to its pediatric-friendly subcutaneous administration and generally favorable safety profile [7,12,84]. Infliximab and adalimumab, monoclonal antibodies that bind both soluble and membrane-bound TNF, are alternative options, particularly for patients with inadequate response to etanercept [37]. The relative efficacy of these agents in DADA2 has not been systematically compared, though most patients who respond to one TNFi respond to others. Lifelong therapy is generally required, as disease flares recur upon discontinuation [85].

Despite the transformative impact of TNFi therapy, several challenges remain. First, as discussed above, the hematologic manifestations of DADA2 are less responsive to TNF blockade than the vasculitic features, reflecting distinct pathogenic mechanisms in the bone marrow compartment. Patients with severe bone marrow failure may therefore still require HSCT despite optimal TNFi therapy [86]. Second, the immunodeficiency component of DADA2 may be unaffected or even exacerbated by TNF inhibition, generating complex risk-benefit calculations in patients with pre-existing hypogammaglobulinemia. Third, the long-term safety of TNFi therapy in pediatric patients with DADA2 is not fully established, though the available data suggest a favorable safety profile. Finally, a minority of patients are refractory to TNFi or develop secondary loss of response, necessitating alternative approaches.

### 7.2. Hematopoietic Stem Cell Transplantation

HSCT is currently the only curative therapy for DADA2, offering the potential for definitive correction of both the inflammatory and hematopoietic defects. The rationale for HSCT is based on the observation that ADA2 is primarily produced by hematopoietic-derived cells, and that engraftment of donor-derived myeloid cells can restore ADA2 enzyme activity and normalize immune function [13,87].

The largest published experience with HSCT in DADA2 comes from a multi-center retrospective study of 30 patients, reporting an overall survival rate of greater than 95%, indicating that HSCT is a definitive cure for DADA2 [88]. Engraftment of donor cells led to normalization of serum ADA2 activity, resolution of the inflammatory syndrome, and improvement in hematologic parameters in the majority of patients. Importantly, HSCT also corrected the immunodeficiency associated with DADA2, with recovery of B cell function and discontinuation of immunoglobulin replacement therapy in most patients [13].

However, HSCT in DADA2 carries significant risks. Transplant-related mortality, graft-versus-host disease, and infectious complications remain substantial concerns, particularly in patients with pre-existing organ damage from stroke or chronic inflammation [89]. The optimal timing of HSCT is a matter of ongoing debate: early transplantation may prevent disease progression and cumulative organ damage, but the morbidity of HSCT must be weighed against the typically excellent response to TNFi therapy in patients with vasculitic-predominant disease. Most experts recommend HSCT for patients with severe hematologic involvement (transfusion-dependent cytopenias, bone marrow failure) or for those with refractory vasculitis despite optimal TNFi therapy [27]. Reduced-intensity conditioning regimens are preferred to minimize toxicity while achieving stable donor chimerism sufficient for ADA2 production [88].

### 7.3. Gene Therapy and Emerging Approaches

Gene therapy represents the next frontier in DADA2 precision therapeutics. The rationale is compelling: a single-gene defect in a predominantly hematopoietic-expressed enzyme is an ideal candidate for ex vivo gene correction in autologous hematopoietic stem cells. Proof-of-concept studies have demonstrated that lentiviral-mediated ADA2 gene transfer rescues ADA2 enzyme activity and corrects macrophage polarization defects in vitro [90]. In mouse models of DADA2, transplantation of gene-corrected HSCs restores serum ADA2 activity, normalizes inflammatory parameters, and prevents vascular pathology [91].

A clinical gene therapy trial for DADA2 is in the planning stages, incorporating lessons from the successful experience with gene therapy for *ADA1* deficiency. Key considerations include the level of ADA2 expression required for clinical benefit, the potential for immunogenicity of the corrected protein, and the optimal gene transfer vector and conditioning regimen [45]. If successful, gene therapy would offer the curative potential of HSCT without the risks of alloreactivity and graft-versus-host disease, representing a major advance in the treatment of DADA2.

Beyond gene therapy, several other therapeutic strategies are under investigation. The JAK-STAT pathway, which mediates signaling from multiple pro-inflammatory cytokines including TNF and type I IFNs, represents a potential target for patients with refractory disease or mixed vasculitic-hematologic phenotypes [92]. Case reports suggest that JAK inhibitors (e.g., ruxolitinib, baricitinib) may be effective in some TNFi-refractory patients, particularly those with prominent interferon signatures. Anti-IL-1 therapy (anakinra, canakinumab) has been tried in a limited number of patients with variable results. Finally, enzyme replacement therapy with recombinant ADA2, analogous to the pegademase approach for ADA1 deficiency, remains a theoretical possibility but has not been clinically developed.

The therapeutic landscape for DADA2 is thus evolving rapidly, from the empirical approaches of the pre-discovery era to the current paradigm of TNFi-first therapy and HSCT for refractory disease, and toward a future of gene therapy and targeted immunomodulation. The key challenge moving forward is not the availability of effective therapies but rather their rational deployment: which patient, at what stage of disease, and with which treatment strategy, will achieve the best long-term outcome? Answering this question will require international collaboration, prospective registries, and a commitment to studying this rare but profoundly instructive disease.

### 7.4. Outstanding Questions and Future Directions

Despite the remarkable progress in understanding DADA2 since its discovery in 2014, fundamental questions remain unresolved. We highlight five key areas in which uncertainty persists and where future research efforts should be directed.

1. Why phenotype divergence? The same ADA2 mutation can cause devastating stroke in one patient and isolated cutaneous vasculitis in another. We speculate that the answer lies at the intersection of genetic modifiers (polymorphisms in TNF, IFN, and adenosine pathway genes), epigenetic reprogramming of the myeloid compartment (trained immunity), and environmental triggers (infectious exposures, microbiome composition). Systematic multi-omics studies of DADA2 discordant sibling pairs—where available—would be particularly informative.
2. Why cerebrovascular tropism? The striking predilection of DADA2 vasculitis for the cerebral circulation remains unexplained. One possibility is that the cerebral endothelium is uniquely dependent on ADA2 growth factor activity for maintaining barrier integrity and that its deficiency creates a site of ‘lowest resistance’ to inflammatory injury [7,23]. Alternatively, the unique adenosine signaling environment of the brain—where adenosine serves as both a neurotransmitter and a vasoregulator—may render the cerebral vasculature particularly vulnerable to ADA2 deficiency. Single-cell transcriptomic comparisons of endothelial cells from different vascular beds in DADA2 patients could reveal the molecular basis of this tissue selectivity.
3. Why is hematologic disease TNFi-resistant? This question has direct therapeutic implications. We speculate that the bone marrow niche is governed by a different cytokine milieu than the peripheral vasculature, with a greater contribution from IFN-γ, adenosine-mediated HSC suppression, and T-cell-derived cytokines. Deciphering the bone marrow microenvironment in DADA2 through spatial transcriptomics and proteomics could identify targets for combination therapy and refine patient selection for HSCT.
4. Is IFN signaling primary or secondary? The presence of a type I IFN signature in a subset of DADA2 patients raises a chicken-and-egg question. If IFN signaling is a primary consequence of ADA2 deficiency (e.g., through impaired clearance of endogenous nucleic acids [24]), then JAK inhibitors may have a role as first-line therapy for these patients. If, however, the IFN signature is a secondary consequence of TNF-driven tissue damage, then effective TNF blockade should suffice. Resolution of this question requires longitudinal profiling of paired TNF-IFN pathway activation in patients before and after initiation of anti-TNF therapy.
5. Enzymatic versus growth factor activity: which matters more? This question goes to the heart of DADA2 pathophysiology. If the vasculopathy is primarily driven by loss of ADA2 growth factor activity, then enzyme replacement therapy (recombinant ADA2) would be predicted to be effective—similar to pegademase for ADA1 deficiency. If, alternatively, the adenosine dysregulation pathway is dominant, then adenosine receptor antagonists or A2B-specific inhibitors may represent a more proximal therapeutic strategy. We speculate that both functions contribute, but their relative importance may differ across tissue compartments and disease stages. Animal models with selective disruption of the enzymatic versus growth factor domains of ADA2 would be invaluable for resolving this question.

To address these questions, we advocate for the establishment of an international DADA2 consortium that integrates: (a) harmonized clinical data collection, (b) multi-omics profiling (genomics, epigenomics, transcriptomics, proteomics, metabolomics) at defined disease stages, (c) functional validation in patient-derived iPSC models and organoids, and (d) a platform for early-phase clinical trials of emerging therapies. Such an integrated approach—modeled after successful consortia for other rare monogenic diseases—would accelerate the transition from description to mechanistic understanding to precision therapy.

## 8. DADA2 as a Model for Monogenic Immune Vasculopathy

DADA2 has emerged as a paradigmatic example of how the study of a rare monogenic disease can illuminate fundamental principles of vascular biology and immunology while also providing a blueprint for precision medicine in inflammatory diseases. Several features of DADA2 contribute to its value as a model system.

First, DADA2 embodies the concept of “one gene, many phenotypes”. The remarkable clinical heterogeneity resulting from mutations in a single gene underscores the importance of modifier factors—genetic, epigenetic, and environmental—in shaping disease expression. Understanding why the same *ADA2* mutation can cause devastating stroke in one patient and mild skin vasculitis in another has implications that extend far beyond DADA2 to the broader question of variable penetrance and expressivity in monogenic disorders [93].

Second, DADA2 illustrates the power of mechanism-based therapy. The identification of TNF as a central pathogenic mediator led directly to the use of anti-TNF therapy, which has profoundly altered the natural history of the disease. This is precision medicine in its most elemental form: understanding the molecular pathology of a disease to select a targeted therapy [94]. The success of this approach in DADA2 serves as a model for how the identification of key pathogenic pathways in other monogenic vasculitis syndromes—such as *STING*-associated vasculopathy of infancy (SAVI) or haploinsufficiency of *A20* (HA20)—can guide therapeutic development.

Third, DADA2 exemplifies the convergence of autoinflammation, autoimmunity, and immunodeficiency within a single disease entity. Traditional immunology has largely treated these categories as distinct, but DADA2 demonstrates that a single genetic defect can produce features of all three [71]. This realization has fostered a reconceptualization of immune-mediated diseases as points on a continuum rather than discrete categories, with implications for the classification, diagnosis, and management of both rare and common immune disorders.

Fourth, the study of DADA2 has unveiled unexpected connections between purine metabolism, endothelial biology, and vascular inflammation. The recognition that a seemingly metabolic enzyme—adenosine deaminase—plays critical non-enzymatic roles as a growth factor and immune modulator has broadened our understanding of moonlighting protein functions [46]. The adenosine signaling pathway is now recognized as a critical checkpoint in vascular inflammation, and ADA2 deficiency has provided a unique window into this regulatory system that may be relevant to more common vascular diseases such as atherosclerosis and hypertension.

Finally, DADA2 demonstrates how genomic discovery translates to clinical practice in rare disease. Gene identification in 2014 led rapidly to evidence-based treatment recommendations through international registries, collaborative consortia, and the repurposing of approved therapies. The lessons learned from DADA2—about the importance of genotype–phenotype correlations, the value of functional validation of genetic variants, and the necessity of multidisciplinary care models—are applicable to the hundreds of other monogenic immune diseases awaiting similar comprehensive characterization.

## 9. Conclusions

The arc of DADA2 research—from bedside to bench and back—exemplifies the transformative power of genomic medicine in rare disease. Yet even this most instructive monogenic model humbles us with fundamental questions still unanswered, a reminder that every layer of understanding reveals new complexity at the interface of immunity, vascular biology, and inflammation. In this sense, DADA2 is more than a model for monogenic vasculitis; it is a proving ground where genetics, immunology, and therapeutics converge to redefine what is possible.

## Figures and Tables

**Table 1:** ADA2 Deficiency: Phenotypic Classification and Corresponding First-Line Treatment Strat egies.

Phenotype Category	Core Clinical Features	Typical Onset Characteristics	Key Laboratory and Imaging Findings	First-Line Clinical Therapeutic Strategy
Vasculitic Phenotype (Most Prevalent)	PAN-like manifestations [4]: livedo reticularis, subcutaneous nodules, digital ischemia, gastrointestinal/renal vascular involvement, testicular pain	Childhood onset (peak 4–6 years) [58], adult onset also documented, predominantly relapsing-remitting disease course	Significantly elevated inflammatory markers (ESR, CRP), inflammatory infiltration of small- and medium-sized arterial walls	Long-term maintenance therapy with TNFi monotherapy as first-line, for rapid control of vasculitis and prevention of disease relapse [7]
Stroke/Neurological Phenotype	Early-onset lacunar infarction, basal ganglia/thalamic infarction, headache, cognitive decline, moyamoya-like vascular changes, cranial nerve palsy [3]	30–50% of pediatric patients present with stroke as the initial manifestation [59,60]; onset ranges from infancy to adulthood	Deep cerebral white matter ischemia, cerebral artery stenosis/occlusion, multifocal chronic ischemic lesions [63,64]	Long-term TNFi-based therapy as first-line, which is the core regimen for preventing de novo stroke and improving neurological outcomes [7]
Hematological Phenotype	PRCA [43], pancytopenia, bone marrow failure, autoimmune cytopenia, Evans syndrome	Predominantly infants and children; may present as an isolated or dominant clinical feature	Bone marrow hypoplasia, dyserythropoiesis, variable degrees of anemia, thrombocytopenia, or neutropenia [35]	HSCT as first-line curative regimen [13]; TNFi is only used as peri-transplant adjuvant therapy for inflammation control
Immunodeficiency Phenotype	Hypogammaglobulinemia, specific antibody deficiency, recurrent infections, B-cell dysfunction, CVID-like phenotype [69,70]	Predominantly childhood onset; severity varies widely	Reduced memory B-cell counts, impaired vaccine response, decreased serum immunoglobulin levels [71]	Intravenous immunoglobulin (IVIG) replacement therapy as first-line; HSCT eligibility evaluation required for severe/progressive cases [11,55]

## Data Availability

No new data were created or analyzed in this study. Data sharing is not applicable to this article.
